# Complete tracheal transection at the thoracic inlet: successful reconstruction with intraoperative Veno-Venous Extracorporeal Membrane Oxygenation (VV-ECMO) support – case report

**DOI:** 10.1186/s12245-026-01162-9

**Published:** 2026-03-11

**Authors:** Huade Luo, Lingling Jiang, Dongying Wang

**Affiliations:** 1 Department of Emergency Intensive Care Unit, Shaoxing Second Hospital, Shaoxing, Zhejiang Province 312000 China; 2 Department of Blood Donation Service, Shaoxing Blood Center, Shaoxing, Zhejiang Province 312000 China

**Keywords:** Tracheal transection, Thoracic inlet, Traumatic tracheobronchial injuries, Veno-Venous Extracorporeal Membrane Oxygenation (VV-ECMO), Case report

## Abstract

**Background:**

Tracheobronchial injuries from blunt chest trauma carry high mortality, with complete thoracic inlet tracheal transection being previously undocumented. While Veno-Venous Extracorporeal Membrane Oxygenation (VV-ECMO) has been increasingly recognized as a critical airway support modality in traumatic tracheal reconstruction, current evidence provides no consensus regarding optimal ECMO initiation timing in surgical decision-making.

**Case presentation:**

A 58-year-old male driver sustained front and back chest impacts in a crash. CT scan revealed complete tracheal transection at the thoracic inlet (mimicking a “bitten-off” appearance). With a dedicated VV-ECMO team on standby, we expeditiously commenced tracheal repair surgery. Intraoperatively, refractory oxygenation deterioration prompted immediate initiation of VV-ECMO via pre-positioned cannulas. Despite this intervention, the patient experienced a 2-minute cardiac arrest, which was successfully reversed. The procedure was ultimately completed with sustained VV-ECMO support, and the patient achieved satisfactory postoperative recovery.

**Conclusions:**

This case demonstrates a unique traumatic tracheal injury mechanism and highlights the critical role of preemptive VV-ECMO team activation in managing complex airway reconstruction.

## Background

Traumatic tracheobronchial injuries (TBIs) resulting from blunt trauma are extremely rare, with a reported incidence between 0.5%-2% [1]. Most lesions (62%-75%) involve the intrathoracic airway, particularly the carina and proximal mainstem bronchi within 2.5 cm of the bifurcation [2, 3]. No prior reports describe complete tracheal transection at the thoracic inlet. Furthermore, Veno-Venous Extracorporeal Membrane Oxygenation (VV-ECMO) has evolved as an important adjunct in TBIs’ surgery over the past decade, and in cases of ineffective oxygenation/ventilation, VV-ECMO support may be used as a salvage therapy perioperatively [4, 5]. We present a novel case of complete tracheal transection at the thoracic inlet following blunt trauma. Despite the preoperative insertion of jugular and femoral venous cannulas with immediate VV-ECMO initiation in case of airway collapse, the patient experienced a brief intraoperative cardiac arrest (2 min), underscoring the critical importance of optimized ECMO timing in airway emergencies.

## Case presentation

**Fig. 1:**
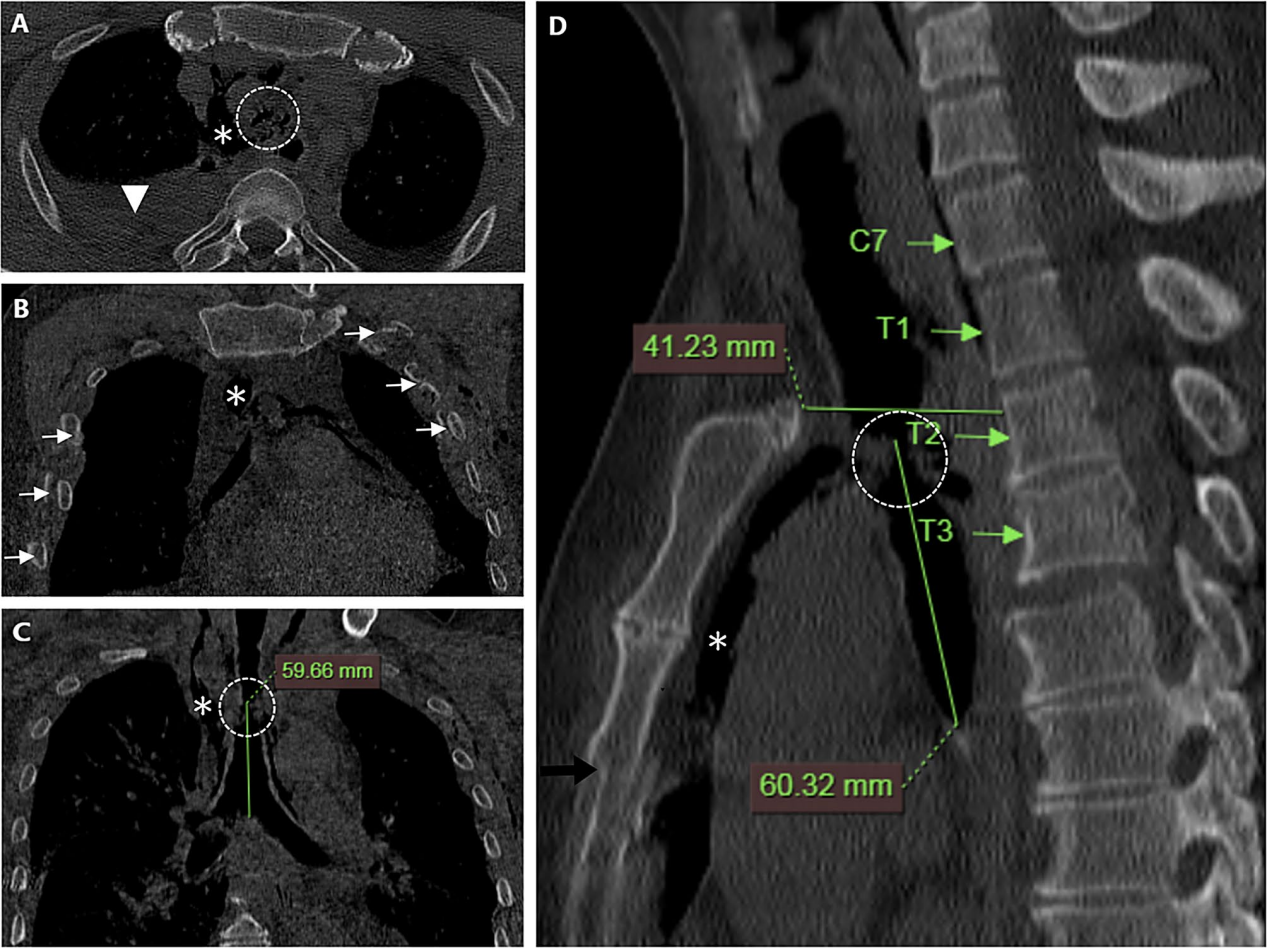
A whole-body CT scan shows a complete tracheal separation 6 cm above the carina (white circle in **A**, **C**, **D**); subcutaneous and mediastinal emphysema (star in **A–D**); right-sided hemothorax (white arrowhead in **A**); sternum fracture (black arrow in **D**); symmetrical bilateral multiple rib fractures (white arrows in **B**); and posterior dislocation of the T3 vertebra (arrow with T3 in **D**). (**D**) The posterior displacement of vertebrae C7 to T3; the shortest distance from the manubrium to the vertebral body is 4.1 cm

A 58-year-old male driver was involved in a crash with emergency braking, which caused the steering wheel and cargo to strike his chest from the front and the back. He remained conscious and complained of chest pain and dyspnea, with muscle strength in both lower extremities at grade 0, and no sensation present. On admission oxygen saturation was measured at 98% with oxygen storage mask 10 L/min, pulse 90 bpm, respiration rate 18/min, and blood pressure at 110/69 mmHg. Physical examination revealed localized dermal abrasions, chest wall depression, and tenderness. The patient exhibited no additional clinical evidence of airway collapse or respiratory decompensation; therefore, endotracheal intubation was not performed at the scene. He was previously healthy, with no comorbidities. A whole-body CT scan revealed a complete tracheal separation 6 cm above the carina, accompanied by subcutaneous and mediastinal emphysema, right-sided hemothorax, sternum fracture, symmetrical bilateral multiple rib fractures, and posterior dislocation of the T3 vertebra (Fig. 1). Fortunately, the enhanced CT scan did not indicate any major vascular injury or intra-abdominal hemorrhage. Following a multidisciplinary trauma team consultation, the decision was made to proceed with thoracotomy for tracheal reconstruction.

Given the potential for difficult intubation and the risk of airway collapse, the anesthesiologist recommended bronchoscopy-guided tracheal intubation to be performed in the operating room and requested that the ECMO team remain on standby as a respiratory support backup in the event of failed intubation. To prepare for potential emergency ECMO deployment, the ECMO team recommends pre-positioning central venous catheters at the VV-ECMO drainage (femoral vein) and perfusion (internal jugular vein) cannulation sites to facilitate rapid insertion of the drainage and perfusion cannulas. However, due to the high cost of materials, the catheters were not unpacked for pre-flushing.

Upon admission to the operating room, the patient underwent ultrasound-guided placement of deep venous catheters in the right internal jugular and right femoral veins. The anesthesiologist performed endotracheal intubation under bronchoscopic guidance, during which an almost circumferential transection approximately 5 cm above the carina was identified, confirming complete tracheal transection. The endotracheal tube was advanced until the tip was positioned near the carina and subsequently secured. Mechanical ventilation was initiated, with no evidence of air leakage and stable oxygenation throughout.

Proceeding via a median sternotomy, the thoracotomy revealed a complete tracheal transection approximately 5 cm proximal to the carina. Unfortunately we observed that the endotracheal tube cuff was positioned at the site of tracheal rupture, with air bubbles emerging from the distal trachea, mixed with blood, significantly impairing the surgical field of view. The anesthesiologist attempted to adjust the intubation depth; however, the attempt was unsuccessful. Meanwhile, the patient exhibited progressively worsening oxygen saturation (SpO2), necessitating frequent suctioning to maintain airway patency. VV-ECMO was urgently initiated to provide respiratory support. Cannulation for drainage and perfusion was rapidly performed via the pre-positioned venous catheters. However, as oxygen saturation could not be maintained, the patient suffered cardiac arrest, and immediate cardiopulmonary resuscitation (CPR) was initiated. Following the establishment of VV-ECMO blood flow, spontaneous circulation was restored. The duration of arrest was approximately 2 min.

Once the VV-ECMO circuit was flowing at 4.3 L/min (3600 rpm) with an FiO₂ of 1.00 and a sweep gas flow of 5 L/min, tracheal repair was performed without systemic anticoagulation. During the procedure, the endotracheal tube was reinserted under direct vision through the glottis, with the cuff positioned to bypass the rupture site and prevent exposure to positive pressure. Due to the severed ends being even and intact and the tracheal stumps being approximable without tension, a circumferential tracheal reconstruction was performed using interrupted 4 − 0 polydioxanone (PDS) sutures, and the anastomosis was subsequently reinforced with a pedicled intercostal muscle flap.

Postoperatively, the patient was transferred to the Intensive Care Unit (ICU). Mechanical ventilation was restarted with a pressure-control ventilator at 40% FiO2 and 5 cmH2O positive end-expiratory pressure (PEEP). We gradually reduced the sweep gas to 2 L/min by the next day, while maintaining the ECMO blood flow at 4.3 L/min (3600 rpm). Given the potential for complications such as ARDS, ECMO was not removed immediately. We progressively reduced the ECMO sweep gas to decrease the level of ECMO support. Because some surface thrombosis was observed in the oxygenator on POD 2, low-dose heparin (125 U/h) was administered for anticoagulation. On POD 4, the patient’s oxygenation index stabilized at approximately 250 mmHg, and ECMO support was successfully weaned. However, the patient developed pulmonary infection, presenting with fever and localized pulmonary consolidation. Following bronchoscopy with secretion suctioning and targeted antibiotic therapy, the patient was extubated on POD 11. On day 29, the patient underwent internal fixation for the thoracic spine fracture and was discharged on day 40 with satisfactory recovery, except for paraplegia.

## Discussion

TBIs typically result from high-energy impacts, and three hypotheses explain their mechanisms [6]. The first hypothesis suggests that excessive pressure in the bronchial tree when the glottis is closed causes airway blowout at the point of greatest diameter-the carina-which occurs most commonly at the junction of the membranous and cartilaginous airway. A second hypothesis is that as the anterior-posterior diameter decreases, the lateral chest diameter increases; consequently, as the lungs are pulled out laterally, the main bronchi and carina become disrupted. According to the third hypothesis, because the carina is tethered, severe and sudden deceleration can give rise to shear forces that may disrupt the airway at points of relative fixation such as the cricoid cartilage and the carina. All three mechanisms indicate that tracheal rupture is most likely to occur around the carina.

In our case, the tracheal transection occurred at the thoracic inlet, and biomechanical analysis of this high-energy impact injury revealed a unique mechanism of tracheal transection. The sudden kinetic transfer during emergency braking precipitated inertial forward displacement of unsecured cargo, generating anterior-posterior compression forces (steering wheel-to-cargo vector) that mechanically compressed the manubrium sterni against T3 vertebra. This resulted in: (1) complete tracheal transection at the anatomical interface of thoracic inlet; (2) symmetrical bilateral rib fractures along anterior axillary lines; (3) posterior vertebral displacement with concomitant spinal canal compromise.

This case delineates a unique mechanism of tracheal injury: unlike classically described carinal ruptures or penetrating by fractures, the trachea suffered complete transection at the thoracic inlet under bidirectional compressive loading (manubrium-to-vertebra vector). This previously undocumented mechanism arises from high-energy crush dynamics distinct from conventional deceleration/shear models.

The severity of injury due to major airway involvement and rapid progression to life-threatening hypoxaemia presents a challenging scenario. The first step in the management of these patients is securing the airway, and the ideal initial management is pre-oxygenation followed by awake flexible bronchoscopy for the evaluation of airway injuries and safe endotracheal intubation [7]. Blind intubation may dislocate fractured cartilage or entirely disrupt a partial tracheal transection, leading to complete airway obstruction [8]. Cross-field ventilation is an important technique in tracheobronchial surgery and requires careful preoperative discussion and coordination between the surgeon and the anesthesiologist.

VV-ECMO has emerged as a viable salvage strategy for maintaining adequate gas exchange during surgical procedures complicated by refractory ventilation failure. The inaugural demonstration of cardiopulmonary support in adult traumatic tracheobronchial injury was documented by Walker et al. in 2012 [9], followed contemporaneously by Enomoto et al.‘s seminal report of perioperative VV-ECMO implementation in a patient sustaining complete tracheal transection 10 mm proximal to the carina [10].

Compared with the lack of experience in cross-field ventilation, ECMO has been increasingly used for life support in critical patients since the outbreak of the COVID-19 pandemic. Our center has also established an ECMO team. Therefore, we chose to use VV-ECMO as a backup in case the patient’s airway collapsed.

Recently, heparin-coated circuits and oxygen membranes have been developed. Short-term heparin-free venovenous ECMO in patients with contraindications to therapeutic anticoagulation could be an effective treatment modality without thromboembolic complications. Maintaining a relatively high ECMO blood flow can also prevent thrombosis [11]. Over the past decade, accumulating evidence has substantiated the expanding utilization of VV-ECMO in the perioperative management of complex tracheal injuries requiring surgical reconstruction [4]. Nevertheless, current clinical guidelines lack consensus regarding evidence-based criteria for optimal ECMO initiation timing in trauma tracheal injury settings.

Given the inherent procedural complexity and time-intensive nature of ECMO initiation, Our team believes that early implementation of ECMO support is warranted in high-risk clinical scenarios of complete tracheal transection. Proactive strategies, including pre-emptive cannulation and rapid mobilization of a dedicated VV-ECMO team, constitute critical components of an optimized emergency response protocol. These measures demonstrably improve significantly increase the success rates of time-sensitive interventions, even in the context of perioperative complications. Evidently, for medical centers with limited experience in bronchial rupture repair, performing tracheal reconstruction under VV-ECMO support represents a prudent and relatively conservative approach.

## Conclusion

A unique “biting-off” mechanism caused by anterior-posterior compressive forces expands the understanding of non-carinal airway injuries. VV-ECMO serves as a critical salvage strategy for oxygenation during surgical repair of traumatic tracheal transection. Standardized pre-emptive cannulation in high-risk cases may mitigate intraoperative deterioration, though significant risks persist.

## Data Availability

No datasets were generated or analysed during the current study.

## Abbreviations

TBIs: Traumatic tracheobronchial injuries
VV-ECMO: Veno-Venous Extracorporeal Membrane Oxygenation
CT: Computed tomography
POD: Postoperative day
